# Using economic analysis to inform health resource allocation: lessons from Malawi

**DOI:** 10.1007/s44250-024-00115-4

**Published:** 2024-07-15

**Authors:** Megha Rao, Dominic Nkhoma, Sakshi Mohan, Pakwanja Twea, Benson Chilima, Joseph Mfutso-Bengo, Jessica Ochalek, Timothy B. Hallett, Andrew N. Phillips, Finn McGuire, Beth Woods, Simon Walker, Mark Sculpher, Paul Revill

**Affiliations:** 1 Centre for Health Economics, University of York Heslington, Alcuin A Block, York, YO10 5DD UK; 2 Health Economics and Policy Unit, Kamuzu University of Health Sciences, Lilongwe, Malawi; 3 Department of Planning and Policy, Ministry of Health, Lilongwe, Malawi; 4 Public Health Institute of Malawi, Ministry of Health, Lilongwe, Malawi; 5 MRC Centre for Global Infectious Disease Analysis, Imperial College London, London, UK; 6 University College London, London, UK

**Keywords:** Resource allocation, Malawi, Health efficiency, Cost-effectiveness analysis, Health benefits packages, Prioritization

## Abstract

Despite making remarkable strides in improving health outcomes, Malawi faces concerns about sustaining the progress achieved due to limited fiscal space and donor dependency. The imperative for efficient health spending becomes evident, necessitating strategic allocation of resources to areas with the greatest impact on mortality and morbidity. Health benefits packages hold promise in supporting efficient resource allocation. However, despite defining these packages over the last two decades, their development and implementation have posed significant challenges for Malawi. In response, the Malawian government, in collaboration with the Thanzi la Onse Programme, has developed a set of tools and frameworks, primarily based on cost-effectiveness analysis, to guide the design of health benefits packages likely to achieve national health objectives. This review provides an overview of these tools and frameworks, accompanied by other related analyses, aiming to better align health financing with health benefits package prioritization. The paper is organized around five key policy questions facing decision-makers: (i) What interventions should the health system deliver? (ii) How should resources be allocated geographically? (iii) How should investments in health system inputs be prioritized? (iv) How should equity considerations be incorporated into resource allocation decisions? and (v) How should evidence generation be prioritized to support resource allocation decisions (guiding research)? The tools and frameworks presented here are intended to be compatible for use in diverse and often complex healthcare systems across Africa, supporting the health resource allocation process as countries pursue Universal Health Coverage.

## Background

In the last two decades, Malawi has made significant progress in health outcomes, evident in a remarkable 10-year increase in life expectancy within the last decade. This notable improvement can be primarily attributed to enhanced well-being of both adults and children, achieved through the effective implementation of lifesaving interventions in HIV, maternal, and child health [1]. Since 2000, Malawi’s Universal Health Coverage (UHC) index has consistently risen, outperforming many peers within the Gross Domestic Product (GDP) per capita range, an outcome policymakers’ credit to strong health system organization [1]. However, significant UHC gaps remain including high under-5 and neonatal mortality rates that are 1.6 times higher than the sustainable development goal (SDG) target; the high prevalence of communicable diseases like HIV/AIDS, Tuberculosis, and Malaria persists; and a rising burden from non-communicable diseases. With per capita health expenditures at just under US$40 per year over the past decade (2012–2021) and projected to increase to only approximately US$49 per capita in 2030 due to severe fiscal space constraints, raising the much-needed revenue for sustaining progress and addressing existing and emerging health challenges remains a serious challenge for Malawi’s health system decision makers. Malawi’s expenditure on health of 9.3% of the total government budget (between 2017 and 2022) is far short of the Abuja Declaration target of 15% [2]. Low domestic health spending and high donor dependency (between 2017 and 2022, donors provided an average of 55% of the total health expenditure (THE), with 24% sourced from the Government, 12% from out-of-pocket expenses (OOP), and the remaining 9% through private health insurance schemes) raises sustainability concerns and the need for enhancing the efficiency of the health system [2, 3].

The limited fiscal space and the urgent need to sustain progress towards UHC underscores the importance of spending existing funds as efficiently as possible. It also emphasizes the need for reorienting health spending and health systems to target diseases and conditions that have the greatest impact on mortality and morbidity. Malawi, like many other LMICs, has tried to achieve this by implementing health benefit packages (HBP) (known in Malawi as the Essential Health Package (EHP)) since 2001 [4]. HBPs most commonly explicitly outline the health care interventions and services to which a country’s population is entitled for free at the point of access, and they increasingly form a central component in the design of health systems [5]. However, determining an HBP requires a number of considerations from policymakers—such as deciding which interventions and programs to include in the package, the series of social objectives they seek to meet (e.g., maximizing population health) in doing so, as well as the constraints in achieving these said objectives (e.g., resources available to fund the HBP). Complexity in HBP design can quickly increase as there might be more than one set of social objectives sought (such as equity or financial protection) or multiple inflexible (in the short run) real-world constraints (human resources or drugs) to the health systems apart from financial constraints that might be of relevance to the policymakers [6]. Such policy decisions around HBP provision are all the more important for a country like Malawi, where the financial, human, and material resources available to provide free and quality healthcare are particularly scarce. Therefore, ensuring an optimally designed and implemented HBP is crucial for a country like Malawi where the opportunity cost of poor resource allocation decisions is substantially high.

In healthcare, economic evaluation, particularly, cost-effectiveness analyses (CEA), is a widely used tool to inform decisions [6]. The principles of CEA inform resource allocation decisions by indicating how limited resources should be allocated to maximize the benefits from their use. In the context of designing an HBP, it can therefore be used to maximize the benefits obtained from all activities funded within the package [6]. This is achieved by weighing the benefits of including a particular intervention against the opportunity costs (i.e., benefits foregone from those other interventions which could instead have been included). In doing so, it applies a systematic and consistent approach to informing priorities [6]. To ensure the practicality and scalability of their upcoming HBPs, Malawi has developed a comprehensive set of tools and frameworks in collaboration with the *Thanzi la Onse* (TLO) Programme (see Box 1), based on CEA, for providing guidance on designing HBPs that align with the nation's health priorities. This paper provides an account of these tools and frameworks, and related analyses for health financing to be better aligned with HBP prioritization.

We have organized the resultant tools and frameworks into five key policy questions facing decision-makers to inform resource allocation: (i) What interventions should the health system deliver? (ii) How should resources be allocated geographically? (iii) How should investments in the health system inputs be prioritized? (iv) How should concern for equity be incorporated into resource allocation decisions? and (v) How should evidence generation be prioritized to support resource allocation decisions (guiding research)? We also identify some major gaps in the underlying methods, data needed to operationalize these tools and frameworks, and suggest ways to improve them to meet policy needs more appropriately in the future. This review provides valuable insights to inform strategy relating to health sector resource allocation and is relevant for all countries pursuing UHC.

Box 1 Thanzi La Onse Programme Since 2019, Malawi has been actively promoting the use of economic evaluation to inform resource allocation decisions through the support of the Thanzi La Onse (TLO) Programme, a partnership between MOH, Kamuzu University of Health Sciences, Eastern, Central, and Southern Africa-Health Community (ECSA-HC), University College London (UCL), Imperial College London (ICL), and the Centre for Health Economics (CHE) at the University of York.

A key element of TLO in Malawi is the establishment of the Health Economics and Policy Unit (HEPU) at the Kamuzu University of Health Sciences. The Unit serves as a hub for strengthening the evidence base locally for responsive health economics and modelling.

## Key policy questions to inform resource allocation

### What interventions should the health system deliver?

**Fig. 1:**
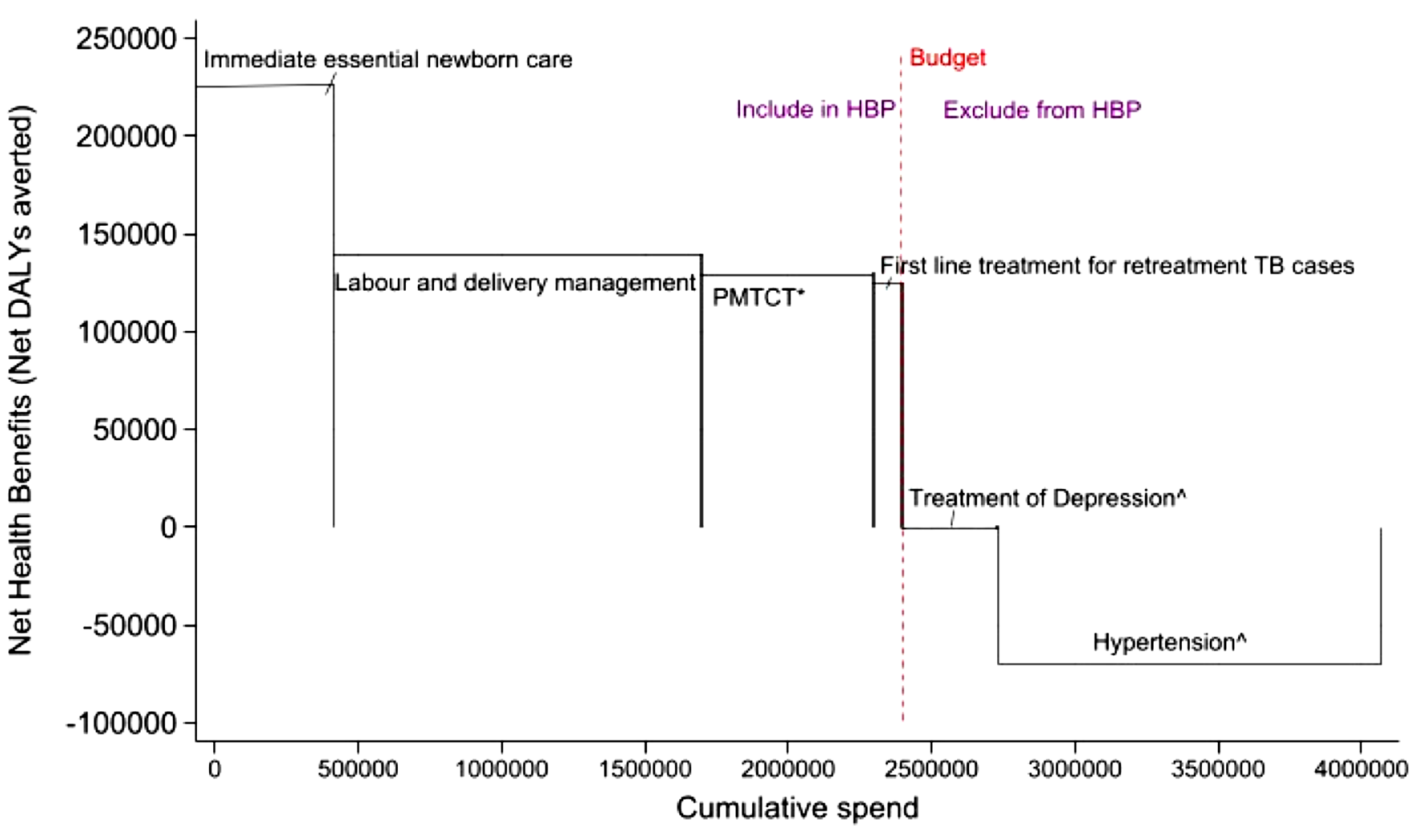
This figure represents a real case study example illustrating how net health benefit can inform the scale of HBP. We selected six interventions from Malawi's EHP, each with varying net health benefits and costs. Based on Malawi’s health opportunity cost of $61 (2015) per DALY averted, interventions on essential newborn care, labour and delivery management, prevention of mother to child transmission (PMTCT-HIV), and first line treatment for retreatment of TB cases (adults), can be included in the EHP as they generate positive net benefits, while interventions on depression treatment and hypertension shouldn’t as they yield negative health benefits. The budget line shows the cumulative spending limit for interventions included in the EHP; exceeding it results in a decline in overall population health. We recognise that the cost-effectiveness database used for developing the EHP (2017) has undergone significant updates since the original publication [12]. With newer evidence available, future decisions regarding the inclusion or exclusion of interventions could be influenced. ^ Hypertension and depression are real and growing problems in Malawi healthcare system especially at primary and secondary levels of care. The Ministry of Health continues to explore ways to provide services for these conditions cost effectively. * PMTCT intervention refers to Option B, a CD4-count based PMTCT program. However, in late-2011, Malawi pioneered the Option B + , a modification of the WHO Option B, which was designed to offer all pregnant and breastfeeding women free lifelong ART at diagnosis, regardless of CD4 count or clinical stage. Subsequently, since 2015, option B + was subsumed in the policy of initiation of ART in all people with diagnosed HIV regardless of CD4 count. Cost effectiveness evidence on this emerged after this research was published. The future EHP revision process should incorporate this new evidence, as it aligns with Malawi's treatment guidelines

For Malawi, currently, the most rigorous process for prioritization of healthcare interventions relates to the EHP, a critical element to achieving UHC, which defines a core set of services to be funded by the health care system to improve service delivery and reduce mortality and morbidity [7, 8]; however, determining these interventions represents a major technical challenge. Malawi’s two previous EHPs, introduced in 2004 and 2011 [9, 10], both promised more than could feasibly be delivered with the available budget. This resulted in interventions being nominally included in the package but unavailable in practice [11]. To overcome this limitation, the EHP was revised in 2017 at MOH’s request, using a new analytical framework [12]. The redesign of the EHP was grounded in the principles of CEA and informed by estimates of the expected net health impact of interventions. An intervention’s net health impact is the health gain it would generate net of its health opportunity cost, where the latter is the amount of health that would otherwise have been generated in the healthcare system using the resources needed to fund the intervention. For healthcare systems aiming to improve population health from within the resources available, estimates of marginal productivity of health expenditure can represent expected health opportunity costs (also known as cost-effectiveness thresholds (CETs)) [13]. This approach advocates that for every DALY averted or QALY gained from a new intervention, the health system should pay no more than the cost at the margin at which it is already able to avert a DALY or gain a QALY from existing interventions. Country-level opportunity-cost-based CETs, as estimated by Ochalek et al. [13] and Woods et al. [14], are available for a wide-range of high-income and low-and middle-income countries (LMICs). What sets net health impact apart from previous approaches used to guide EHP design in Malawi [4, 5] is that it accounts not only for the per-patient cost and health benefit (i.e., cost-effectiveness) of each intervention, but also the size of the patient population that stands to benefit and the opportunity cost associated with each intervention. As such, this measure guides prioritization decisions more directly and more clearly align decisions with the promotion of overall population health, than other measures used previously [12] (Fig. 1).

In Malawi, the framework was used to inform the content and scale of the EHP given existing resources and the value of expanding the coverage of EHP services, through funding implementation efforts specific to interventions and/or health system strengthening which may improve the implementation of a range of interventions. A detailed description of how the framework was applied in Malawi’s context is provided by Ochalek et al. [12]. Though the analyses were conducted using the best available evidence in Malawi, there are limitations both in terms of comprehensiveness and quality of the available evidence. Another important limitation is that the analysis does not consider interdependencies between interventions, assuming their costs and effects remain independent of each other, and costs and health effects are linear in implementation. Despite this, the framework was able to guide the revision process successfully. The review process for Malawi’s 2017 EHP involved two steps. First, estimates of the net health impact of interventions and potential combinations of interventions were generated using the framework. Then, a consultative process around package design was conducted to deliberate and finalise the interventions for the EHP based on the evidence provided by the analysis, incorporating expert opinion and consensus building with stakeholders from district health offices and tertiary hospitals (rather than to prescribe a particular package or level of overall health expenditure). Interventions for which there was limited cost-effectiveness data were assessed for inclusion during the second consultative phase.

The outcome of the deliberative process, which took into account additional considerations such as equity, continuum of care, complementarity of services, and exceptional donor-funded interventions alongside net health impact, resulted in Malawi's final Essential Health Package (EHP) for 2017. This package comprised a positive list of 97 interventions, with an estimated annual cost of $247 million and predicted to avert 41.5 million DALYs if fully implemented. Key interventions, deemed as "best buys", include those targeting HIV prevention, TB prevention and treatment, maternal and neonatal health, and malaria prevention and treatment. Similar to its predecessors, the cost of this package exceeded the budgeted resources (by about 73%). However, if fully executed, it would cost 31% less than the 2011 package ($362 million) while averting 92% as many DALYs. Consequently, it presented a more realistic and less aspirational package in comparison [11].

Twea and colleagues [4] outline Malawi's deliberative process for developing, implementing, and monitoring the 2017 EHP. Their paper documents that despite the expectation for providers at the service delivery level to prioritize EHP interventions, the system of free healthcare access in Malawi poses challenges in restricting non-EHP interventions. This is particularly difficult when centrally procured resources can be used interchangeably for both EHP and non-EHP interventions. Consequently, these challenges led to further analysis aimed at aligning public financial management mechanisms with EHP prioritization [4]. This included revising the geographical resource allocation formula, conducting bottleneck analyses to identify health system gaps for under-performing EHP interventions, carrying out regular resource mapping exercises according to the EHP, and conducting EHP distribution impact analyses. Vertical program management proved to be another additional challenge in implementing the EHP [4]. Currently, Malawi employs a minimalist approach to prioritize EHP services nationwide, but more robust approaches could be implemented through a comprehensive revision of purchasing processes. Overall, the implementation of the EHP has enhanced the monitoring and evaluation system for public health services in Malawi, with improved reporting linkages for community healthcare workers [4].

### How should resources be allocated geographically?

There is a growing consensus on the benefits of defining an HBP in countries' journeys towards UHC [8]. However, the effective realization of UHC goals necessitates not only the optimal allocation of limited healthcare resources among various health services, but also across different geographical areas. In the context of Malawi's decentralized healthcare system, these geographic areas correspond to its districts. With a total estimated population of 17.6 million people, Malawi is divided into 28 districts, where about 84% of the population resides. The rest reside in the country’s four cities. The Ministry of Local Government & Rural Development (MoLGRD) oversees healthcare delivery at the district level through its district health councils and is responsible for the allocation of the drugs and other recurrent transactions (ORT) budgets to the districts. The personal emoluments (e.g., salaries) and development (e.g., capital expenditures) budget are managed by other central government departments.

To distribute the recurrent transactions budget, a Health Resource Allocation Formula (HRAF), part of a broader Intergovernmental Fiscal Transfer formula, was used. Unfortunately, weak implementation and inconsistent updates to the formula lead to a HRAF that sustains historical allocations, resulting in each district receiving the same relative shares of the budget. However, in 2018, during the early stages of HSSP-II implementation, driven by the demand from district councils for more equitable and needs-based funding, the MOH began the technical work to develop a sub-national RAF for the health sector, but this is also yet to be adopted and implemented [15].

**Fig. 2:**
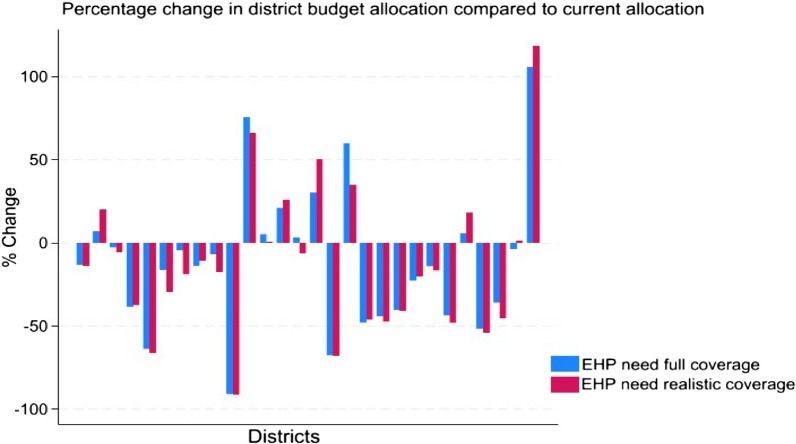
This figure represents the percentage shifts in district allocation from current to EHP need-based formulas

Unlike other needs-based formulae applied in LMICs [16, 17], Malawi’s 2018 health sector RAF aimed to explicitly align with its EHP [18]. Such a linkage is necessary to bridge the disconnect between the EHP design, the national and subnational resource allocation processes, and EHP implementation at the service provider level. Moreover, the emphasis on EHP coverage as a priority for the MOH indicated its significance as a cornerstone for developing the RAF [15]. Therefore, using demographic, epidemiological, and health sector budget data, three versions of a resource allocation formulas were proposed to gradually incorporate more details on factors influencing the expected annual expenditure on the EHP provision in each district [18]. Essentially, the revised RAF is developed based on an estimate of the projected cost of implementing the EHP for each district and allocates resources proportionally according to need. The cost-of-service delivery is calculated by considering the unit cost of providing each intervention, along with the expected number of cases based on incidence, prevalence, and utilization rates. This RAF design enables resource allocation to align with the expected level of service delivery [4]. Detailed descriptions of each formula, the data used, and the subsequent findings can be found in McGuire et al. [18]*.* The formulas suggest significant changes in budgetary allocations for most districts compared to the allocation in 2018 (Fig. 2). In certain instances, the magnitude of these shifts exceeded 50%, either as reductions or doubling of district budgets [18]. The results emphasize that certain districts exhibit a notably higher demand for EHP-related healthcare services, regardless of their population sizes, due to variation in EHP-related disease burdens. Findings also show that some districts with lower EHP-related disease burdens and populations are receiving comparatively high allocations under the current district allocation system. This could reflect other factors like genuine differences in healthcare delivery costs, targeted donor funding, or potential biases in allocation influenced by differences in political power. These substantial adjustments underscored inequities in the previous budget allocation system and highlighted the potential for significant improvements.

The study by McGuire and colleagues recognizes that there are data-related and practical challenges in implementing such a resource allocation formula. It outlines approaches for addressing certain key limitations encountered during the formula’s development, such as the absence of consideration for donor allocations, receipts from other sources, funding beyond district drug and ORT budgets, and the inclusion of information on inter-district migration.

The proposed RAF was presented in extensive stakeholder consultations while maintaining district anonymity to ensure discussions remained focused on the objectives and components of the formula and not its implications for individual districts. Twea and colleagues [15] discuss in detail Malawi’s experience in 2019 to legally adopt and implement this new RAF. It is likely that the immediate implementation of the proposed RAF would be logistically and politically infeasible, especially on account of the substantial recapitalization needed to make implementation feasible [15]. Without this funding, implementation timelines are spread out over a substantially longer timeframe, rendering their implementation ineffective. Nonetheless, Malawi’s RAF revision process underscores the advantages of linking sub-national funding to nationally defined HBPs and demonstrates how such linkage can serve as a suitable initial measure to guarantee the effective utilization of existing resources [15, 18]. It also highlights the need to support such revisions with financial commitments to make the RAF operational.

### How should investments in the health system inputs be prioritized?

Conventionally, the design of an HBP is informed by the principles of CEA – i.e., it is centered around supporting decisions to enhance population health gains from the services included in the package, while simultaneously taking into account other priorities, like equity, all within a given financial constraint, as demonstrated in Malawi’s 2017 EHP [6]. CEA can also be applied using constrained optimization methods [6, 19]. Understandably, budgetary restrictions are not the only constraint on the health system. In the short term, there exist diverse constraints on care, both financial and non-financial (e.g., staffing, drug availability) [20–23]. Thus, it is essential to showcase how additional resource constraints can be incorporated alongside the public health budget constraint, offering analytical support for decisions concerning HBP [23]. Under such circumstances, the simple CEA approach can be augmented to include additional considerations. This not only allows policymakers to explicitly account for multiple constraints when determining health services coverage, but also assess the incremental (marginal) value associated with easing explicitly modelled health systems constraints [24].

Connolly and colleagues demonstrate the application of a linear constrained optimization approach (LCOA) [25] in guiding the design of an HBP using Malawi’s latest EHP ahead of the third Health Sector Strategic Plan (HSSP 2023–2030) [1]. A recent systematic review of literature on economic evaluation of health system strengthening (HSS) [26] in LMICs reveals that previous studies have primarily concentrated on assessing how HSS enables the delivery of interventions within specific disease areas or programs. The LCOA approach would be the first to produce empirical evidence on the value of HSS (i.e., relaxing health system constraints) across a broad spectrum of health interventions.

The LCOA-based framework maximizes the net DALYs averted (or net health impact) given the financial and physical resource constraints of Malawi’s public health sector [23]. The size of the resource envelope allocated for purchasing drugs and consumables represents the financial constraint, while the size of the health workforce, including doctors, clinical officers, nursing staff, and pharmaceutical staff, represents the physical constraint. A total of 141 interventions were considered for inclusion in the Malawian EHP. The LCOA was used to determine the cost-effective combination of interventions and optimal coverage levels that would maximize net DALYs averted [6]. Based on the LCOA, Malawi’s HBP included 39 interventions in the baseline scenario, averting 51.7 million net DALYs in total [25]. Delivering these 39 interventions exhausts the doctor/clinical officer capacity; however, the consumable budget (i.e., drugs and commodities) and remaining health worker capacity remain underutilized. Additional scenarios were also run using the optimization model. One scenario focused solely on the drug budget constraint, excluding the health workforce constraint. This resulted in a HBP comprising 59 interventions, averting a total of 55.1 million net DALYs. In another scenario, donor constraint was considered alongside the drug budget constraint, while excluding the health workforce constraint, leading to an optimal package of 66 interventions averting 53.4 million net DALYs [25]. Further, the marginal value analysis, mirroring Mohan and colleagues’ [23] analysis for Uganda, suggested that the most important bottleneck in the Malawian Health system is the shortage of doctors/clinical officers, which makes it difficult to further expand the HBP. More specifically, $1000 invested towards buying additional doctor/clinical officer time could result in over 275 net DALYs averted. In other words, the incremental cost-effectiveness ratio of hiring additional doctor/clinical officer time is $3.6 per DALY averted, net of opportunity costs.

Although it was possible to apply the approach in a setting with relatively limited data, such as Malawi, replication of these data-intensive methods in other contexts would need to take account of the extent and quality of available evidence. Despite these challenges, the framework showed how investments in health system strengthening (HSS) can provide high value by ameliorating health system constraints that limit the delivery of interventions. To ensure criteria beyond cost-effectiveness and resource constraints were considered, the LCOA was combined with a participatory MCDA to consider societal values and preferences, such as severity of illness, effectiveness, poverty reduction, vulnerable populations, and level of care. Following both the constrained optimization and MCDA analyses, experts from the government, funders, implementing partners, and civil society came together to agree on the interventions to be included in the final EHP [25].

To support the operationalisation of HBP in Malawi, several reforms and changes in service delivery are in the planning phase as part of the HSSP III [25]. Pooling government and donor budgets along with joint development of implementation plans based on HSSP III activities is being undertaken to improve the alignment of nonfungible donor funding with HBP priorities. Another significant reform under HSSP III is transitioning from vertical disease programs to integrated service delivery platforms to advance the implementation of HBP for UHC attainment [25].

Additionally, forthcoming studies from Malawi are set to enhance the body of evidence guiding health system investments, specifically in supply chain strengthening. Mohan and colleagues [27] will be focusing on exploring facility-level characteristics associated with consumable availability across multiple disease programs in their upcoming work. Concurrently, Mangal and colleagues [28] will be projecting the potential impact of stockouts of essential medicines on the achievement of targets within the HIV or TB programs.

### How should concern for equity be incorporated into resource allocation decisions?

The design of HBPs allows for the selection of interventions based on explicitly defined objectives. Malawi’s 2017 EHP revision process demonstrated how aggregate population health outcomes can be considered in determining a package within a given budget. However, apart from improving health outcomes, another key consideration for policymakers in Malawi is ensuring equity in health, access to healthcare, and utilization [29–31]. Notably, equity considerations were informally factored into the EHP design due to insufficient evidence on the impacts of interventions on health inequality [31].

Health inequality impacts can be evaluated alongside population health using distributional cost-effectiveness analysis (DCEA) [32]. Usually, a full DCEA approach requires bespoke estimates of health benefits and costs from a decision analytic model or trial-based analysis [32, 33]. However, in contexts with limited data, such as Malawi, using this method to measure the distributional impacts of intervention can be challenging. In such cases, a less data-intensive approach, called the aggregate-level DCEA [32], serves as an alternative to assess the proportion of the overall population net health benefits attributable to various equity-relevant population groups for each intervention. It allocates population level health benefits to equity-relevant groups based on their share of healthcare utilisation for the targeted disease [31–33]. This permits the selection of interventions based on their potential to improve population health and diminish health inequity [31]. To showcase the viability of this approach, Arnold and colleagues [31] utilized aggregate DCEA to shed light on the equity impact of Malawi’s 2017 EHP. This approach takes incremental costs and health benefits reported from the database of cost-effectiveness evidence that was established to inform the Malawian EHP [12] and combines these with socioeconomic distributions of utilisation (from publicly available surveys and census data) and opportunity cost to estimate how the net health benefit from an intervention is divided among equity relevant subgroups within the population[31]. Early applications of DCEA have been limited to high-income countries [34] or to evaluations of individual interventions [35]. This study is the first to employ an aggregate DCEA to demonstrate how potential health package interventions differ in their impact on health inequality and population net health [31]. It was motivated by the practical challenges faced in the implementation of the 2017 EHP in Malawi [4] and originated during the early stages of HSSP-II.

Results from the analysis show that 70% (36 out of 51 interventions included in Malawi’s current EHP) of the interventions are health improving and inequality reducing. At the same time, 7.8% (4 out of 51) of the interventions in the EHP have negative population net health benefit and increase health inequality. Furthermore, the EHP prioritizes interventions that are not only more accessible to the poor but also address conditions that are more prevalent among this demographic. However, the current patterns of EHP access do not benefit the poorest households the most [31]. Interventions with substantial health benefits, such as active management of the third stage of labour, first-line tuberculosis treatment, and the management of obstructed labour, are still underutilized by the poorest households. Realizing full utilization of the EHP could lead to the greatest gains for the poorest households [31].

Making such frameworks useful to support policy depends on the extent and quality of available evidence. Moreover, the socioeconomic characteristics considered for the analyses might not be ideal for capturing Malawi’s health inequality concerns, which ideally should reflect the views of policymakers. In spite of these challenges, the above results demonstrate the feasibility of utilizing aggregate DCEA to underpin the development of HBPs in two significant ways: 1) by facilitating formal consideration of inequality impacts, and 2) by offering a systematic and reasoned approach for assessing any trade-off between impacts on health inequality and overall population health [31].

### How should evidence generation be prioritized to support resource allocation decisions (guiding research)?

The success of Malawi’s EHP in achieving its objectives is dependent on the availability of robust evidence to guide resource allocation decisions. Published cost-effectiveness (CE) studies, which were used to inform the set of interventions to be provided through the Malawi EHP form an uncertain evidence base. This raises the question of whether research to generate additional evidence to resolve this uncertainty could improve HBP design. Approaches proposed and used in the past to quantify uncertainty in the cost-effectiveness of interventions (e.g., stochastic league tables, a method where interventions are ranked based on their probability of being cost-effective [36]) are unable to adequately answer these questions [37]. Consequently, research strategies may not be aligned with the evidential needs of jurisdictions in defining their HBPs [37]. Value of information (VOI) methods have been recommended to support HBP design and research prioritization [37, 38]. The limited use of these methods in the past in this area may be a result of their technical complexity. To overcome these barriers, Schmitt and colleagues [37] presented a simplified framework and a companion tool designed to assess the areas where additional evidence would be most impactful in producing health gains, using only the information that typically informs HBPs [37]. Specifically, it uses the sensitivity analysis results reported in the cost-effectiveness literature to produce probability distributions of incremental costs and incremental health benefits, based on which it derives a probability distribution of population net health impact and, using VOI analysis, generates estimates of the population net health impact from research expressed in DALYs. There is currently no other framework known to us that illustrates the integration of VOI principles with HBP design for prioritizing research fund allocation [37].

Applying this to the evidence base underpinning the design of Malawi’s 2017 EHP, Schmitt and colleagues demonstrate that by addressing the uncertainty regarding which healthcare interventions are most likely to effectively enhance net population health when funded, research has the capacity to refine HBP design [37]. This, in turn, holds the potential to yield additional health gains. A detailed description of the approach and the theoretical foundation can be found in Schmitt et al. [37]. Out of the 67 interventions considered for Malawi’s HBP, the framework was applied to a sub-set of 21 interventions. Results highlighted three interventions as healthcare research priorities: ‘voluntary male circumcision (VMMC),’ ‘community management of acute malnutrition in children,’ and ‘isoniazid preventive therapy in HIV + individuals no TB.’ Investing in research to inform future investments in these interventions has the potential to avert approximately 122,000 net DALYs over a 20-year time span [37].

Naturally, like other methods, this approach also hinges on the availability of CE evidence and accompanying sensitivity analyses. Additionally, it is also important to explore different modes of implementation to enhance the cost-effectiveness of interventions. In the case of VMMC, for example, Bansi-Matharu and colleagues [39] demonstrate that provision of VMMC for 5 years, followed by re-evaluation and potentially withdrawing its availability, is likely to be cost-effective in many African settings. Such evidence could influence the establishment of research priorities.

Moreover, internationally, current research prioritization processes do not commonly incorporate the kind of evidence discussed here. However, there are ongoing institutional changes in Malawi to enable the adoption of VOI methods [38]. It is expected that the framework described above will offer important quantitative insights to support the recently established Health Technology Assessment (HTA) Task Force which will oversee institutionalization of HTA in Malawi including the identification of research priorities to inform the design, implementation, and monitoring of HBP. Additionally, it can also provide meaningful input to deliberative research prioritization processes that take into account a broader range of social objectives in conjunction with health outcomes [37]. Fundamentally, these contributions play a pivotal role in guiding significant policy considerations regarding the distribution of research funds in settings with limited resources [37].

## Other developments and analyses

In this section, we outline some additional novel analyses that have been undertaken in Malawi to answer policy questions in allocating healthcare resources beyond those mentioned above.

### Evaluating a program with multiple stakeholders (inter-sectoral allocation)

**Fig. 3:**
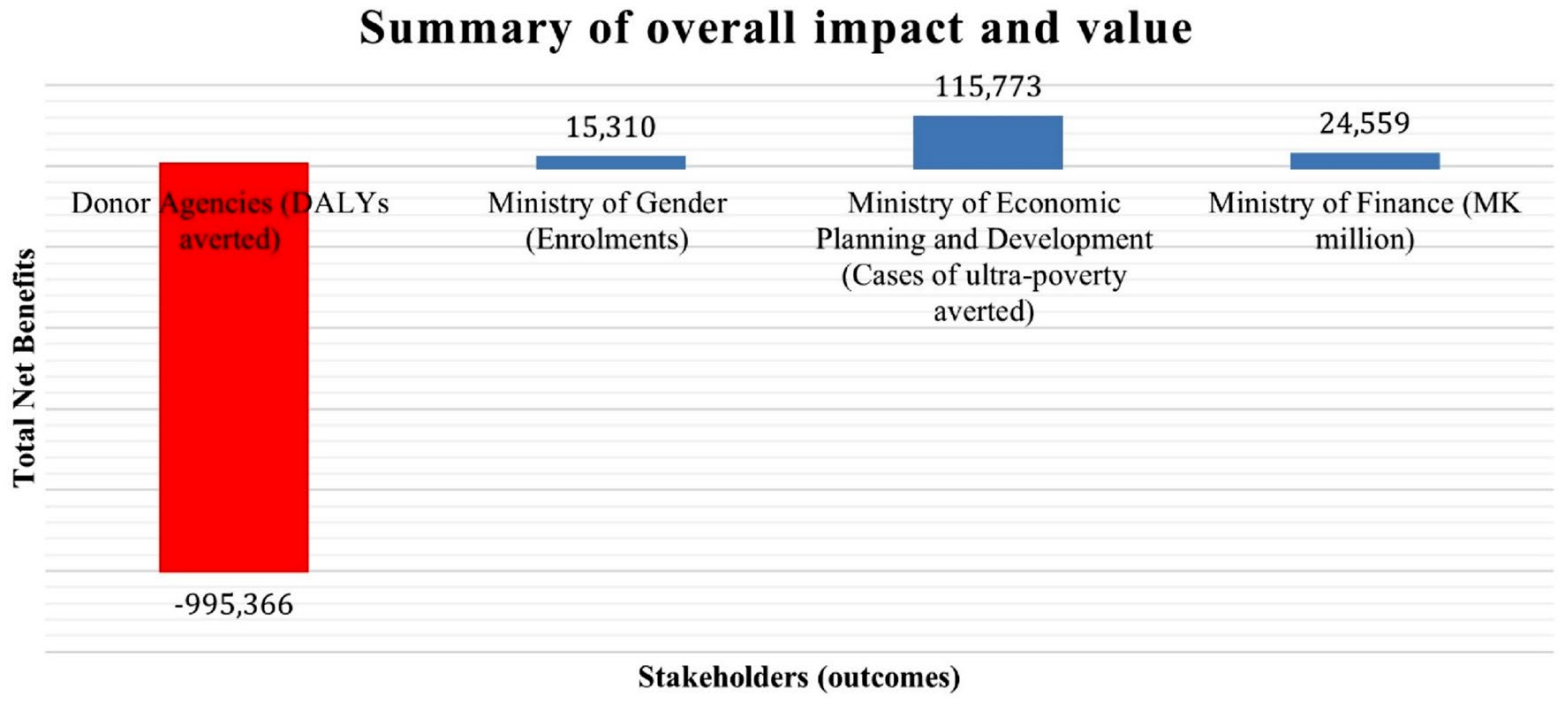
This figure presents the net benefits (obtained as direct effects net of opportunity costs) according to the perspective of each of the stakeholders involved in Malawi’s SCTP

Over the last two decades, there has been a heightened policy focus on recognizing the impact of sectors beyond healthcare in determining population health [40, 41]. Even at the national level, health is increasingly recognized as an outcome jointly generated with non-health sectors’ actions [42]. In Malawi, especially, numerous programs (e.g., nutrition; cash transfer) with significant health impacts, entail collaboration among diverse stakeholders spanning various sectors of the economy. Naturally, the different stakeholders participating in the program will have distinct scope and objectives and their assessment of the program’s impact may vary [43–45]. In such situations, an economic evaluation that takes a perspective that is relevant to a single decision maker may be unsatisfactory if costs, savings or (dis)benefits fall on others [46–48]. The ‘societal perspective’ sometimes advocated generally ignores the reality of multiple decision-makers with different responsibilities, objectives, and budget constraints. To overcome this gap in economic evaluation methods, Walker, and colleagues [49] proposed an analytical framework to present costs, savings and (dis)benefits of relevance to multiple decision-makers, the trade-offs between them and how this can support complex multi-sector decisions. Ramponi and colleagues [50] illustrate the first field testing of this framework by conducting a cross-sectoral economic evaluation of the social cash transfer program (SCTP) in Malawi. The SCTP is described in terms of the dimensions of its value (defined by a selected outcome measure such as DALYs averted for health), and the populations of interest (sub-groups directly or indirectly affected (through opportunity costs) by the policy), relevant to stakeholders involved in its implementation. The dimensions, outcomes and population groups contribute to a structured table known as an impact matrix. Once populated, this matrix allows for the information to be summarized from various perspectives, elucidating the value judgments necessary to combine impacts across dimensions and population groups [49]. Further details about the applied framework are available in the study conducted by Ramponi and colleagues [49]. Results indicated that although the SCTP provided value for money from the standpoint of national stakeholders, the same wasn't true for international donors (Fig. 3). When viewed through the lens of an international donor, the health opportunity costs incurred by forgoing other investments internationally are not outweighed by the health benefits experienced by the recipients of SCTP in Malawi. In simpler terms, allocating funds to the SCTP leads to fewer DALYs averted compared to if had been used for other purposes internationally, leading to a net failure in averting 995,366 DALYs for the broader population. Although the use of such methods is presently in infancy, its application to the cash transfer programme in Malawi open doors for extensions to other interventions outside the health sector which have implications on health such as education [50, 51].

### A whole-health-system' perspective for identifying value-for-money investments in health system strengthening

In order to understand how the delivery of services can be organized to enhance efficiency now and, in the future, the interacting economic and epidemiological processes by which health gains are generated needs to be understood. Thus, health planning may benefit from full health system modelling that captures the interplay of healthcare system capabilities, population knowledge, access, and use of healthcare. Such a *‘whole system and all-diseases’* model (the Thanzi La Onse (TLO) model [52]) is being developed for Malawi. It aims to capture the health experiences of the Malawian population and the consequent interactions with the healthcare system. Built with a modular disease epidemiology design, the Malawi TLO model simulates longitudinally the lives of a representative sample of the Malawian population, and represents a number of key health conditions, and corresponding diagnoses and treatments in the areas of reproductive, maternal, newborn, and child health, communicable and non-communicable diseases, which jointly represent the majority of the disease burden in Malawi. The model mimics the population’s demographics, risk factors, experience of disease, encounters with the healthcare system, and the effects on its health of the healthcare received. It explicitly represents the generation of health gains in a population, which can be used to examine the effect of resource allocation, management, and clinical practice, to inform decision-making. By explicitly modelling multiple disease areas, resource constraints and healthcare-seeking patterns, the TLO model effectively places the patient care pathway at the center of policy evaluation. It does this by capturing the interrelations between health conditions and the efficacy of treatment strategies and allowing for the propagation of cross-cutting effects or indirect consequences of any intervention. A more in-depth description of the model can be found at [https://www.tlomodel.org/]. Results from such a model will provide valuable input to the development of the medium-term and long-term policy setting in Malawi, including updates to the EHP, HSSP, and strategies for healthcare system strengthening.

## Future research priorities

This paper presents novel frameworks and tools designed to respond to some of the most pressing resource allocation questions facing the Government of Malawi (see Table 1). These have the potential to be applied more widely in Africa and in other settings to align national decision-making with local priorities. However, there is considerable scope to improve the evidence and methods underpinning the frameworks in the future and to meet policy needs more appropriately.

**Table 1:** Summary of all tools and frameworks developed to inform health resource allocation in Malawi

Relevant policy question	Tools or frameworks	Impact
What interventions should the health system prioritise for deliver?	Ochalek et al. 2018 [12]: an analytical framework grounded in the principles of CEA and informed by estimates of the expected net health impact of interventions that explicitly accounts for the health opportunity costs associated with an intervention	The analysis informed Malawi’s 2017 EHP comprising a positive list of 97 interventions, which cost 31% less annually than the 2011 package while averting 92% as many DALYs
How should resources be allocated geographically?	McGuire et al. 2020 [18]: a new framework for the district health RAF based on districts’ relative EHP-related need in Malawi	The resultant district health RAF suggested significant changes in budgetary allocations for most Malawi districts, sometimes exceeding 50%, either as reductions or doubling of district budgets. The National Local Government Finance Committee has initiated deliberations for the RAF’s legal adoption and implementation in Malawi
How should investments in health system inputs be prioritised?	Connolly et al. 2024 [25]: Linear Constrained Optimisation approach (LCOA) that maximises net health impact given the financial and non-financial resource constraints of the health system, demonstrating where investments in the health workforce offer greatest health returns	Delivering the proposed revised EHP would exhaust doctor or clinical officer capacity, while leaving the consumable budget and remaining health worker capacity underutilized. This suggests that in Malawi investing in expanding doctors/clinical officer capacity as a key form of HSS is vital for greater health gains
How should concerns for equity be incorporated into resource allocation decisions?	Arnold et al. 2020 [31]: AGGREGATE distributional cost effectiveness analysis (DCEA)	Analysed equity implications of the EHP. Results showed that 70% of the interventions included in Malawi’s 2017 EHP were health improving and inequality reducing. Although the current EHP prioritizes interventions used more by the poor, they do not reap the greatest benefits. This suggests that inequality effects require greater consideration in priority setting and resource allocation
How should evidence generation be prioritised to support resource allocation decisions?	Schmitt et al. 2021 [37]: analytical framework and companion tool based on Value of information (VOI) methods to show which uncertainties in the evidence base have greatest potential health consequences	Identified ‘male circumcision,’ ‘community management of acute malnutrition in children,’ and ‘isoniazid preventive therapy in HIV + individuals no TB’ as key research priorities for Malawi with a potential to avert more than 122,000 net DALYs over 20 years. It is expected to inform Malawi’s recently established HTA Task Force in research prioritisation
How to evaluate a program with multiple stakeholders?	Ramponi et al. 2022 [49]: analytical framework for economic evaluation when costs and benefits fall on multiple sectors and decision makers	Illustrated that Malawi’s social cash transfer program provided value for money from a national stakeholder perspective but not from the viewpoint of a donor interested in health outcomes. This could inform discussions about alternative funding arrangements or prompt donors to adopt a broader perspective that health alone
How to identify value for money investments in health system strengthening?	Hallett et al. 2023 [52]: a novel individual-based modelling approach spanning most diseases and health services delivered by the Malawi healthcare system	Allows you to simulate the long-term effects of the system-level interventions in Malawi

First, CEA comparing a fuller range of alternative interventions relevant to the African context is needed to provide policymakers with more complete evidence for various resource allocation decisions. Alternatives include different modes of delivery, integration with different sets of services and different target populations. Consulting policymakers prior to designing primary research and cost-effectiveness studies to identify the policy choice set can be a useful way to ensure the relevance and comprehensiveness of studies.

Second, going forward, it is hoped that the MOH and its partners will establish suitable processes for building the capacity to conduct CEA and generate evidence within Malawi. This involves a shift away from dependence on secondary sources derived from healthcare systems that may exhibit significant differences from Malawi. Notably, the MOH has already taken steps in this direction by establishing a taskforce responsible for HTA institutionalization to aid cost-effective decision-making [53]. Indeed, the HBP framework can help to prioritize where new evidence for Malawi would be most valuable. Moreover, the government-funded HEPU at Kamuzu University can act as a potential research-to-policy channel in strengthening this capacity and ensuring that HTA is a success in terms of research and analysis informing real decision-making in a timely manner.

Third, the HBP revision process in a country often assumes a blank slate from which to start. Current methods ignore the transition costs of removing and adding new interventions including staff training, updating provider payment mechanisms and dissemination of revised intervention guidelines. Future studies should aim to capture these transition costs explicitly. Also, the current application of HBP design methods assumes perfect flexibility in allocation of health system resources. In reality, however, resource constraints operate at a sub-national level and are either completely rigid or moveable at a cost. Accounting for sub-national constraints will allow for HBP design to be closer to reality.

Fourth, to improve evidence on health system strengthening (HSS), the constrained optimization approach utilized in Malawi could be replicated for other settings and modelled for a fuller range of health system inputs (e.g., of supply chains, pre-and in-service training, etc.).

Fifth, the current methods assume constant marginal cost and effects of interventions at different levels of coverage and in combination with different services. The ‘whole system and all-disease model’, like the one in Malawi, could help relax this assumption by allowing for the incorporation of economies of scale and scope. However, this will be dependent on the generation of more detailed evidence. Further, HBP design within the context of the whole system and all-disease models can seek to capture the dynamic epidemiological effects and costs of various HBP intervention choices.

The utility of these tools and frameworks extends beyond the five policy questions discussed in this paper. Decision-makers in LMICs also grapple with questions concerning the delivery and enhancement of the quality and effectiveness of Health Benefit Package (HBP) services. Moving forward, we anticipate that these methods can be expanded to explore these additional areas in greater detail.

Last, experience shows that healthcare resource allocation is not merely a technical exercise but is also a political choice. These frameworks should take cognizance of the influence of political forces and be complemented with other approaches that explore the political economy aspects during budgeting, resource allocation, and priority setting [54].

## Conclusion

The above review shows how Malawi has embraced economic evaluation methods, especially, CEA as a formal research tool to support decisions about resource allocation in healthcare in general, and the development of HBP in particular. This is also reflected well in the latest HSSP-III 2023–2030 [1] and health sector financing strategy 2023 [55], which shows a continued commitment to using economic analysis, in particular economic evaluation, in policy making. While there may have been other tools suitable for addressing each policy question, we have exclusively covered those that were actually utilized in Malawi. Based on the recognition of the existence of resource constraints and the resulting opportunity costs of directing these limited resources towards an intervention or investment, the tools and frameworks reviewed allow for the efficient allocation of physical and financial resources to attain country objectives. When applied together, these offer a comprehensive approach to effectively guide the resource allocation process and sustain progress towards UHC. Given the many competing demands on fiscal resources, evidence of value for money for investing in health matters for ministries of finance and the development community alike and will strengthen health ministries’ ability to advocate for a greater share of the budget in the future.

## Data Availability

Not applicable.
